# Clinical Course of Transgender Adolescents with Complicated Postural Orthostatic Tachycardia Syndrome Undergoing Hormonal Therapy in Gender Transition: A Case Series

**DOI:** 10.1089/trgh.2019.0041

**Published:** 2019-11-20

**Authors:** Jeffrey R. Boris, Zachary B.R. McClain, Thomas Bernadzikowski

**Affiliations:** ^1^ Pediatric Cardiologist, Independent, Media, Pennsylvania.; ^2^ Division of Adolescent Medicine, Children's Hospital of Philadelphia and Perelman School of Medicine, University of Pennsylvania, Philadelphia, Pennsylvania.; ^3^ Division of Radiology, Children's Hospital of Philadelphia, Philadelphia, Pennsylvania.

**Keywords:** dysautonomia, LGBT health, orthostatic intolerance, testosterone therapy

## Abstract

**Purpose:** Postural orthostatic tachycardia syndrome (POTS), an increasingly recognized dysautonomia, may affect as many as 3,000,000 Americans. Concurrently, prevalence estimates suggest 10% of individuals identify as lesbian, gay, bisexual, transgender, or questioning/queer. The preponderance of female POTS patients implies hormonal differences between natal sexes and their role in POTS. Transgender POTS patients using hormone therapies may offer further insight into the mechanism of POTS. There have been no previously published studies of transgender patients with POTS undergoing gender-affirming hormone therapy.

**Methods:** We reviewed our electronic health record for clinical histories of transgender patients in our POTS Database.

**Results:** Three patients who transitioned from female to male demonstrated clinical improvement of their POTS symptoms with the addition of testosterone therapy.

**Conclusion:** We present our clinical experience of three transgender POTS patients who transitioned from female to male with hormone therapy, all of whom demonstrated clinical improvement with testosterone. This may give further insight into the pathophysiology of POTS. However, the authors do not endorse the use of hormone therapy as primary therapy for the symptoms of POTS.

## Introduction

Postural orthostatic tachycardia syndrome (POTS), a dysautonomia that is increasingly being recognized and diagnosed, is thought to affect as many as 3,000,000 people in the United States.^1,2^ At the Children's Hospital of Philadelphia (CHOP) alone, nearly 950 pediatric patients through age 18 years were diagnosed from 2007 to 2018. Prior publications have reviewed demographics of this population of patients, including a 3.45:1 female to male ratio as well as an overwhelming preponderance of Caucasian patients.^3,4^ Concurrently, prevalence estimates show that ∼10% of individuals in the general population identify as lesbian, gay, bisexual, transgender or questioning/queer (LGBTQ).^5^ Caring for LGBTQ youth is a part of every general pediatric practice,^6^ and thus gender diverse and transgender adolescents may be of particular interest within this group of patients with POTS.

In the context of gender diverse or transgender youth, it is necessary to first understand the difference between natal sex and gender identity. An individual's natal sex is the sex identification assigned at birth. Natal sex is most frequently determined by external genitalia, but may also take into account chromosomes, reproductive organs, or hormone levels. Alternatively, an individual's gender identity is their internal sense of who they are. There is a wide range of gender identities, including female, male, a combination of both, somewhere in between, or neither. Individuals predominantly have a gender identity that matches their natal sex; cisgender is the term used to describe these individuals. For some individuals, their natal sex and gender identity do not align, and they are referred to as gender diverse or transgender.^7^ In the Diagnostic and Statistical Manual of Mental Disorders (DSM-5), people whose sex designated at birth is contrary to the one with which they identify and is accompanied by distress or impairment from this incongruence, are diagnosed with gender dysphoria.^8^

There are little data regarding the role of hormones in the pathophysiology of POTS. The preponderance of female to male POTS patients raises questions regarding hormonal differences between natal sexes and the role these may play in how POTS is expressed clinically, with natal females possibly at greater risk or having more sensitivity to autonomic perturbations. Transgender POTS patients, especially those using hormone therapies in their transition, may offer further insight into the mechanism of POTS. There have been no previously published studies of transgender patients with POTS undergoing gender-affirming hormone therapy. The clinical experience of three transgender POTS patients in our patient population, all of whom were transitioning from female to male with hormone therapy, is presented in this study.

## Cases

Patient 1 is a natal female identifying as male (uses he/him/his pronouns) who was diagnosed with POTS in November 2014 at age 16 years. He had a 1 year history of dizziness and headaches, with subsequent onset of fatigue, cognitive dysfunction, insomnia (both in getting and staying asleep), early satiety, numbness, joint pain, heat and cold intolerance, exercise intolerance, dyspnea with activity, diaphoresis, palpitations, tachycardia, photophobia, and hyperacusis, and with the clinical findings of venous pooling, joint hypermobility, easy bruising, dislocation, and hyperelastic skin. Past medical history was notable for hypermobile Ehlers–Danlos syndrome, exercise-induced asthma, and overweight. At diagnosis, his medications included cyclobenzaprine, ibuprofen, and naproxen; he had allergies to latex, cetirizine, kiwi, and nuts. Family history was notable for Ehlers–Danlos syndrome in his mother, brother, maternal aunts, maternal uncles, and maternal cousins. On examination, resting blood pressure was 122/80 mmHg. On 10-min stand, heart rate persistently went from 95 to 135 beats per minute. He was noted to have joint hypermobility and venous pooling, but an otherwise normal examination and ECG. He was treated with cyproheptadine for headache prophylaxis, fludrocortisone for dizziness, and pregabalin for pain—his three worst symptoms. The headaches resolved, but his dizziness persisted, despite attempts at therapy with fludrocortisone, midodrine, and desmopressin. His pain did not respond to pregabalin or duloxetine. The pain was severe enough to prevent routine exercise to suppress POTS symptoms.

He was seen in the Gender and Sexuality Clinic at CHOP 1.5 years after his diagnosis of POTS. He was diagnosed with gender dysphoria, and started testosterone therapy for hormonal management of female to male gender-affirming therapy. Subsequent testosterone levels have been in the low-normal physiological male range. In November 2018, 4 years after diagnosis with POTS and 2 years after initiation of testosterone, he had no complaints of headaches or dizziness, and was off of all blood pressure supportive therapies. His blood pressure was elevated at 140/80 mmHg, and was treated with metoprolol. He continued to have pain, however, which was attributed to the joint hypermobility associated with Ehlers–Danlos syndrome.

Patient 2 is a natal female identifying as male who was diagnosed with POTS in March 2017 at age 17 years. He had a 5 year history of dizziness with symptom onset after menarche, and with subsequent onset of blurry vision, syncope (numerous events), fatigue, cognitive dysfunction (improved with Strattera), insomnia (specifically getting to sleep, which improved with trazodone), headache, nausea, diarrhea, constipation, early satiety, abdominal pain, numbness, muscle pain, joint pain, heat intolerance, dyspnea with activity, chest pain, palpitations, tachycardia, photophobia, hyperacusis, and with the clinical findings of venous pooling, joint hypermobility, easy bruising, and hyperelastic skin. Past medical history was notable for attention deficit disorder with hyperactivity, bipolar disorder, exercise-induced asthma, hypermobile Ehlers–Danlos syndrome, and obsessive-compulsive disorder. At diagnosis, his medications included albuterol, quetiapine, atomoxetine, and trazodone. He had no allergies. Family history was notable for autistic spectrum disorder, but no other pertinent disorders. On examination, resting blood pressure was 116/65 mmHg, with heart rate persistently increasing from 70 to 105 on 10 min stand. Joint hypermobility and venous pooling were noted, with no other significant clinical findings. He was treated with cyproheptadine for headaches, fludrocortisone for dizziness, and pyridostigmine for early satiety—his three worst symptoms. He was also referred for physical therapy for education for joint protection and stabilization as well as for use of an exercise protocol for POTS. Headaches and early satiety improved with medical therapy, and he started routine exercise. Attempts to control dizziness were unsuccessful, despite therapy with fludrocortisone and desmopressin. Increased pyridostigmine dosing for persistent constipation led to muscle twitching, so the dose was reduced.

He was seen in the Gender and Sexuality Clinic in February 2019, 2 years after POTS diagnosis. He was subsequently diagnosed with gender dysphoria and started on testosterone therapy. In March 2019, resting blood pressure was 117/72 on low dose midodrine, with a heart rate increase from 72 to 102 beats per minute on 10 min stand. He also continued on cyproheptadine, pyridostigmine, and salt capsules for POTS therapy.

Patient 3 is a natal female identifying as male who was diagnosed with POTS in July 2015 at age 16 years. He had a 10 year history of migraine, with subsequent onset of dizziness, blurred vision, fatigue, cognitive dysfunction, insomnia (both getting to and staying asleep), occasional nausea, early satiety, abdominal pain, numbness, muscle pain, joint pain, heat intolerance, cold intolerance, exercise intolerance, dyspnea with activity, diaphoresis, chest pain, palpitations, tachycardia, photophobia, and hyperacusis, and with the clinical finding of venous pooling. Past medical history was notable for migraine, anxiety disorder, obsessive-compulsive disorder, depression, Hashimoto's thyroiditis, bicuspid aortic valve with trivial aortic valve insufficiency and no stenosis, and juvenile idiopathic arthritis. Medications at diagnosis included albuterol, azelastine, diclofenac, fludrocortisone, fluoxetine, fluticasone/salmeterol, folic acid, gabapentin, levothyroxine, melatonin, methotrexate, salt tablets, ranitidine, tramadol, and zolmitriptan. He had no allergies. Family history was notable for fibromyalgia, but no other significant disorders. On examination, resting blood pressure was 104/72, with a persistent heart rate increase of 70–140 beats per minute on 10 min stand. Venous pooling was noted, but the remainder of the examination demonstrated no abnormalities. He was started on desmopressin for dizziness, lansoprazole for symptoms of gastroesophageal reflux, and methylphenidate for fatigue—his three worst symptoms. He also started exercising regularly with an exercise protocol for POTS. Dizziness was persistent, and required increased desmopressin and subsequent addition of midodrine. With regular exercise, symptoms improved. Stimulant therapy was subsequently discontinued in 2016. However, in February 2017, he sustained a concussion, which worsened symptoms of dizziness and fatigue, limiting his ability to exercise regularly.

In May 2017, he was seen in the Gender and Sexuality Clinic, was diagnosed with gender dysphoria, and started testosterone therapy. Subsequently, his symptoms associated with POTS, including dizziness, insomnia, fatigue, headache, and cognitive dysfunction, all significantly improved by report. Interestingly, he reported that when he had gaps in testosterone therapy, his POTS symptoms would return. In March 2019, resting blood pressure was 122/68 on midodrine and desmopressin as blood pressure supportive therapy. He was exercising regularly and had only rare dizziness with no complaints of palpitations, dyspnea, chest pain, or syncope. The therapeutic plan going forward included discontinuing the desmopressin. Testosterone levels were in the normal physiological male range subsequent to starting supplementation.

## Conclusion

POTS is a dysautonomia that leads to multiple disabling symptoms. Pediatric patients with POTS often are not able to attend school or participate in typical activities of daily living.^9^ Although described >25 years ago,^10^ the pathophysiology of POTS remains undefined. There are data suggesting various triggers, such as infection and concussion,^4^ a high incidence of associated joint hypermobility,^4,11,12^ that autoantibodies are increasingly being found in association with POTS patients,^13,14^ and that natal females are more affected than natal males.^15,16^ However, none of these have been clearly demonstrated to be the singular etiology or a contributing factor to an abnormal pathway that leads to this disorder. Natal female sex preponderance in the disorder is yet another observation that may offer additional insight into how POTS symptoms manifest, and the potential effect of hormones. In this report, we discuss the clinical course of three transgender patients with POTS as natal females identifying as males who subsequently started testosterone for gender-affirming therapy. All three patients demonstrated apparent clinical improvement in their POTS symptoms with addition of supplemental testosterone. This improvement was independent of the use of medications to reduce their symptoms, and actually led to a reduction in the need for medications to control symptoms associated with POTS. Typical therapy for POTS includes high amounts of fluid and salt intake, exercise to suppress symptoms, and, as needed, medications to control symptoms so that patients can be functional and maintain the ability to exercise.^17,18^ Before initiation of hormone therapy, the symptoms associated with POTS in all three patients showed variable control, with overall improvement after addition of these interventions. However, these patients also demonstrated that symptom control drastically improved after testosterone was added. Notably, by patient report, when one of our patients would miss supplemental testosterone therapy, symptoms of POTS would subjectively worsen.

Some caveats need to be understood in the interpretation of this case series. First, the patients' POTS symptoms were not evaluated with an objective measure of symptom severity, so all reports of symptomatology were subjective. Second, and more importantly, this case series is not a recommendation that patients with POTS should utilize male hormones to try to reduce symptoms. The reductions in symptomatology were unexpected additions to the primary therapeutic goal, namely hormonal gender-affirming therapy from female to male for these transgender adolescents. Certainly, hormone therapies, such as fludrocortisone^18,19^ and desmopressin,^18,20^ are used in treatment of the symptoms of POTS. These are not, however, gender-related therapies, nor do they have gender modifying effects. The recognition that male hormone therapy in these patients may have influenced the reduction of POTS symptoms supports the observation of natal female sex predominance in those patients who are symptomatic. It may be that females and males are actually affected more equally with the underlying pathophysiology, but that females demonstrate more severity of, or decreased relative tolerance to, symptoms. It also may be that natal females are more “susceptible” to the disease process that leads to POTS. Further attention should be given to LGBTQ patients who have concurrent POTS and use hormonal gender-affirming therapy, to try to further define if and how their symptoms of POTS improve with therapy.

## Abbreviations Used

CHOP: Children's Hospital of Philadelphia
POTS: postural orthostatic tachycardia syndrome
